# Isolation of Anti-Prion Compounds from *Curcuma phaeocaulis* Valeton Extract

**DOI:** 10.3390/molecules29174034

**Published:** 2024-08-26

**Authors:** Jaehyeon Kim, Hakmin Lee, Hye Mi Kim, Ji Hoon Kim, Sanghoon Byun, Sungeun Lee, Chul Young Kim, Chongsuk Ryou

**Affiliations:** Department of Pharmacy, College of Pharmacy and Institute of Pharmaceutical Science and Technology, Hanyang University ERICA, Ansan 15588, Gyeonggi-do, Republic of Korea; rlawoguses@hanyang.ac.kr (J.K.); gkrals92@hanyang.ac.kr (H.L.); hyemi586@hanyang.ac.kr (H.M.K.); gg890718@gmail.com (J.H.K.); chok9916@hanyang.ac.kr (S.B.); guranye@hanyang.ac.kr (S.L.)

**Keywords:** prion diseases, *Curcuma phaeocaulis*, curcumenol, furanodienone, anti-prion agents

## Abstract

Prion diseases, known as a group of fatal neurodegenerative disorders caused by prions, remain incurable despite extensive research efforts. In a recent study, crude extract from *Curcuma phaeocaulis* Valeton (*Cp*) showed promising anti-prion efficacy in in vitro and in vivo models, prompting further investigation into their active compounds. We endeavored to identify the chemical constituents of the *Cp* extract and discover potential anti-prion agents. With the use of centrifugal partition chromatography (CPC), major constituents were isolated from the *n*-hexane (HX) fraction of the extract in a single step. Spectroscopic analysis confirmed the presence of curcumenone, curcumenol, and furanodienone. Subsequent efficacy testing in a cell culture model of prion disease identified curcumenol and furanodienone as active compounds. This study underscores the potential of natural products in the search for effective treatments against prion diseases.

## 1. Introduction

Prion diseases are a group of rare fatal neurodegenerative disorders caused by unconventional proteinaceous pathogens called prions [1]. They include Creutzfeldt–Jakob disease in humans and scrapie, bovine spongiform encephalopathy, and chronic wasting disease in animals [2]. Prions are composed of a misfolded isoform of cellular prion protein (PrP^C^), termed scrapie prion protein (PrP^Sc^) [3]. Formed by the conformational conversion of PrP^C^, PrP^Sc^ begins to accumulate in the brain [4] where it directly and indirectly triggers neurotoxicity, leading to neuronal cell death, vacuolation, and spongiform degeneration [5].

Currently, prion diseases are incurable. Although no treatment in clinical use to ameliorate the course of prion disease is available [6], numerous agents from different classes have been investigated using in vitro and in vivo models [7]. Natural extracts and natural product-derived compounds also belong to these agents. Representative anti-prion agents obtained from natural extracts include baicalein from *Scutellaria lateriflora* L. [8], carnosic acid from *Rosmarinus officinalis* L. [9], curcumin from *Curcuma longa* L. [10], polydatin from *Polygonum cuspidatum* Siebold & Zucc. [11], cannabidiol from the *Cannabis* genus [12], hinokitiol from the *Cupressus* genus [13], epigallocatechin gallate from green tea [14], ginsenoside-RG3 from ginseng [15], resveratrol from a variety of fruits like grapes and berries [16], and trehalose from almost all organisms [17].

*Curcuma phaeocaulis* Valeton (*Cp*), a plant species in the Zingiberaceae family, is commonly found in East Asia and has been used as traditional herbal medicine to control conditions related to blood stasis, gastritis, and pain [18]. Phytochemical research has identified various bioactive constituents in *Cp*, particularly sesquiterpenoids, which exhibit a range of therapeutic properties for cancer [19], inflammation [20], and neurotoxic conditions [21]. *Cp* is also noted for its potential use in treating neurodegenerative diseases [22]. Specifically, several sesquiterpenoids in the *Curcuma* genus were reported to improve pathophysiological molecular and cellular events associated with Alzheimer’s disease [23,24,25,26] that shares similarities with prion disease in terms of neurodegeneration caused by protein misfolding and aggregation.

In our recent study, we reported the anti-prion efficacy of crude *Cp* extract [27]. The in vitro assay results demonstrated that *Cp* extract inhibited conversion and aggregation of misfolded PrP. Additionally, they suppressed prion infection in cultured neuroblastoma cells and the accumulation of PrP^Sc^ in the cultured neuroblastoma cells with chronic prion infection. Furthermore, the crude extract was effective in treating prion disease in a mouse model, showing prevention of disease onset, astrogliosis and spongiosis in the brain, as well as PrP^Sc^ accumulation in the brain and spleen in nearly 40% of prion-infected mice in the group.

It is important to identify the compounds of the *Cp* extract that result in beneficial efficacy. The aim of the current study is to determine the chemical constituents of the *Cp* extract and discover active anti-prion compounds in this extract. Separation and purification of bioactive molecules from natural products often require repeated chromatography, and sometimes the active ingredients may not elute due to absorption onto the solid phase. By enhancing separation efficiency and sample recovery, these challenges can be overcome through counter-current chromatography, such as centrifugal partition chromatography (CPC), which is a valid alternative [28,29]. CPC, a liquid–liquid chromatography technique utilizing two immiscible solvent phases, is an efficient tool for separating bioactive components from natural products based on their biological activity. It offers advantages such as a higher sample loading capacity, no sample loss due to irreversible absorption onto solid columns, flexible operation modes, and the ability to choose from a variety of solvent systems [30].

In this study, we isolated major constituents from the *n*-hexane (HX) fraction of the *Cp* extract using CPC in a single step. The isolated compounds were confirmed to be curcumenone (**1**), curcumenol (**2**), and furanodienone (**3**) using spectroscopic methods (MS and NMR). Finally, these compounds were applied to efficacy testing in a cell culture model of prion disease. This study successfully identified curcumenol (**2**) and furanodienone (**3**) as active compounds. 

## 2. Results and Discussion

### 2.1. Activity-Guided Identification of Active Fractions in the Cp Extract for Anti-Prion Activity 

**Figure 1:**
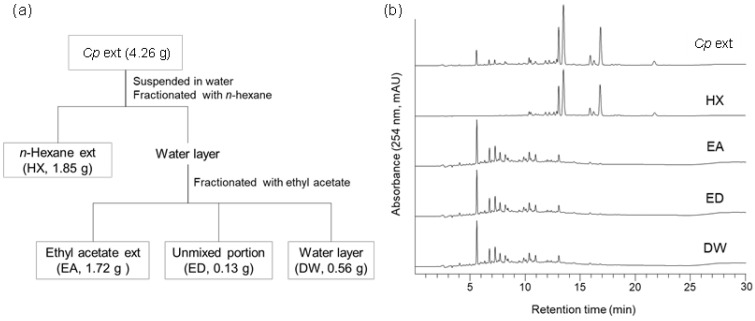
Solvent fractionation of crude *Cp* extract. (**a**) Fractionation scheme. (**b**) HPLC chromatograms of crude extract and sub-fractions. HX, *n*-hexane; EA, ethyl acetate; ED, unmixed portion of EA and DW fractions; DW, distilled water.

**Figure 2:**
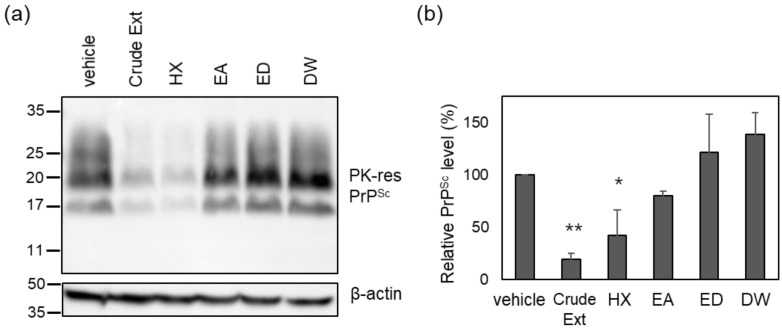
The level of PK-resistant PrP^Sc^ in the cells incubated with solvent-extracted *Cp* fractions. (**a**) A representative Western blot of PK-resistant (res) PrP^Sc^. The crude *Cp* extract was evaluated for anti-prion activity at a concentration of 50 μg/mL, and each solvent-extracted fraction was used to incubate ScN2a cells at the concentration of assigned weight ratios as in Table S1. (**b**) Densitometry analysis of PK-resistant PrP^Sc^. *n* = 4. * *p*-value < 0.05; ** *p*-value < 0.01.

To identify the active compounds in the crude *Cp* extract responsible for prion suppression described in a previous report [27], solvent fractionation was conducted using *n*-hexane (HX), ethyl acetate (EA), and water (DW) (Figure 1A). The major peaks found in the *Cp* extract were present in the HX fraction (Figure 1B). Subsequently, each fraction was used to incubate cultured neuroblastoma cells with chronic prion infection (ScN2a cells) according to the weight ratio of each fraction (Table S1). The level of PrP^Sc^ in ScN2a cells incubated with the HX fraction, but not the others, decreased to a comparable level observed for the crude extract (Figure 2). The anti-prion activity of the crude *Cp* extract shown in the current study was reminiscent of that reported in a previous study [27]. Although the anti-prion activity of the HX fraction and the crude *Cp* extract did not appear to be the same, the difference was not statistically significant. Taken together, the results indicate that the HX fraction retains the most activity to inhibit PrP^Sc^ accumulation in cultured cells, which was shown by the crude extract. 

### 2.2. Isolation of Major Compounds from the Cp Extract by CPC

To reveal the active components of the crude extract, we aimed to separate the major peaks observed in the HPLC chromatogram of the extract. As mentioned earlier, since the major peaks of the extract were found in the HX fraction and this fraction exhibited anti-prion activity (Figure 1B and Figure 2), the HX fraction was used for further experiments to separate the major peaks.

Successful separation of target compounds by CPC necessitates the selection of an appropriate two-phase solvent system. To optimize CPC solvent conditions, the *K*-values of the major constituents in various solvent systems with different compositions and volume ratios of two immiscible solvents were evaluated (Table S2 and Figure S1). Based on this result, the solvent system HX:ethanol:water (10:7:3) exhibited the most effective separation for major constituents (Table 1). The *K*-values of curcumenone (**1**), curcumenol (**2**), and furanodienone (**3**) were 0.26, 0.58, and 2.14, respectively.

**Figure 3:**
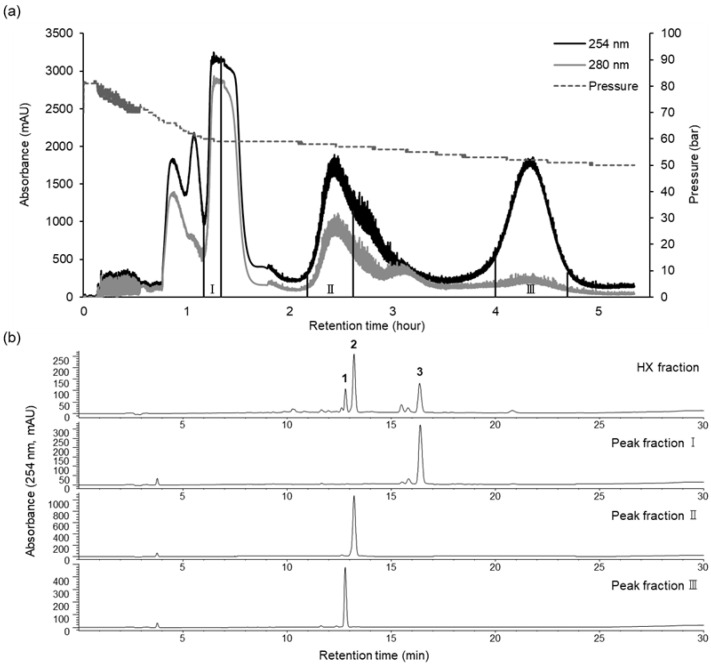
CPC chromatogram of the HX fraction (**a**) and HPLC analysis of CPC peak fractions I–III (**b**). CPC conditions: two-phase solvent system, *n*-hexane:ethanol:water (10:7:3, *v*/*v*/*v*); ascending mode; flow rate of 10 mL/min; rotation speed of 1200 rpm; monitored at 254 (black line) and 280 (gray line) nm. HPLC conditions are the same as those described above.

The preparative CPC operated in the ascending mode, with the lower phase as the mobile phase and the upper phase acting as the stationary phase. During the process, 4.7 g of HX fraction was fractionated in a single run of 330 min, the retention of the stationary phase in the coil was 69.5%, and the pressure was 82 bar. The preparative CPC chromatogram is shown in Figure 3. Analysis of the HPLC peak area showed that fractions II and III contained purified compounds (curcumenol (**2**) and curcumenone (**1**), respectively) of up to 90% (Figure 3), with 190.1 and 914.9 mg of curcumenone (**1**, 97.78%) and curcumenol (**2**, 94.80%), respectively, isolated and collected from 4.7 g of the HX fraction. CPC peak fraction I was further subjected to preparative HPLC using an isocratic mode, and furanodienone (**3**, 99.28%) was obtained with high purity.

### 2.3. Determination of Chemical Identity

**Figure 4:**
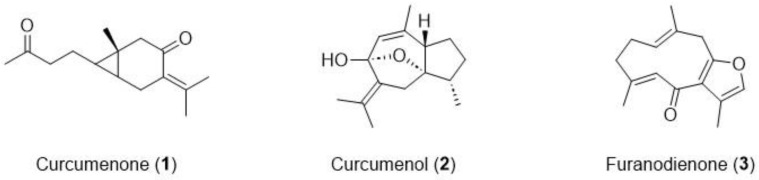
Chemical structures of the isolated compounds from *Cp* extract.

The structural identification of CPC peak fractions (I–III) was carried out by ESI-MS, ^1^H-, and ^13^C-NMR (Figures S2–S8). In comparison to the reported data [31], the chemical structures of peak fractions I–III were elucidated as curcumenone (**1**), curcumenol (**2**), and furanodienone (**3**), respectively (Figure 4). Interestingly, none of these compounds shares chemical identity with curcumin, a compound widely found in the *Curcuma* genus and previously reported to inhibit PrP fibrilization and PrP^Sc^ accumulation in vitro [10].

### 2.4. Evaluation of the Anti-Prion Activity of the Major Compounds That Constitute the HX Fraction

**Figure 5:**
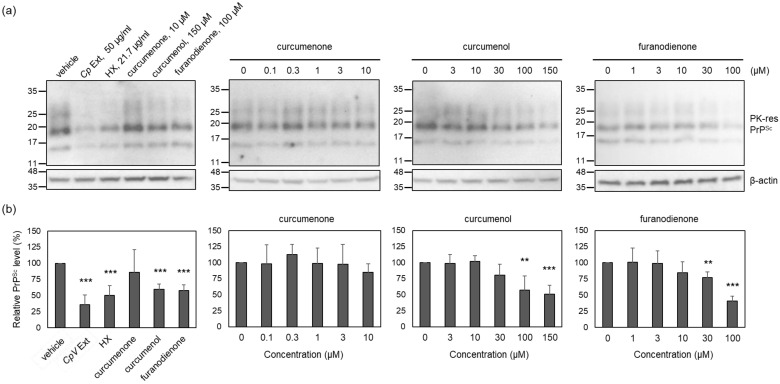
The level of PK-resistant PrP^Sc^ in the cells incubated with major compounds that constitute the HX fraction. (**a**) Representative Western blots of PK-resistant (res) PrP^Sc^. Cells were incubated with non-cytotoxic concentrations or below for each compound, HX fraction, and crude extract. (**b**) Densitometry analysis of PK-resistant PrP^Sc^. *n* = 4. ** *p*-value < 0.01; *** *p*-value < 0.001.

To estimate the anti-prion activity of identified compounds included in the active HX fraction, the PrP^Sc^ level was measured in ScN2a cells incubated with non-cytotoxic concentrations of curcumenone (**1**), curcumenol (**2**), and furanodienone (**3**). Non-cytotoxic concentrations were determined by MTT assay. The results showed that ScN2a cells survived without cytotoxicity at and below 10 μM for curcumenone (**1**), 150 μM for curcumenol (**2**), 100 μM for furanodienone (**3**), 50 μg/mL for the crude *Cp* extract, and 22 μg/mL for the HX fraction (Figure S9). At the highest non-cytotoxic concentrations, the level of PrP^Sc^ was reduced by curcumenol (**2**) and furanodienone (**3**), but not by curcumenone (**1**), when compared to that found in the vehicle control (Figure 5). This reduction was comparable to the effect exhibited by the HX fraction, a positive control. The anti-prion activity of curcumenol (**2**) and furanodienone (**3**) occurred in a concentration-dependent fashion, although complete elimination of PrP^Sc^ was infeasible within the non-cytotoxic concentrations. These results suggest that the efficacy of the crude *Cp* extract in modulating prion propagation in cells and animals appears to be attributed to the anti-prion activity exerted by curcumenol (**2**) and furanodienone (**3**).

Although curcumenol (**2**) and furanodienone (**3**) each alone significantly reduced the PrP^Sc^ levels, the activity was insufficient to represent that of the crude *Cp* extract. This makes us speculate that the combination of two active compounds could be more efficient to suppress prion propagation than each compound alone. Thus, reconstitution of these two active compounds would imply the accomplishment of full anti-prion activity demonstrated by the crude extract.

## 3. Materials and Methods

### 3.1. Apparatus and Materials

Centrifugal partition chromatography (CPC) was carried out using an SCPC-100+1000 equipped with a Spot prep II LC system (Armen Instrument, Saint-Avé, France). HPLC analyses were performed using an Agilent 1260 Infinity HPLC system (Agilent Technologies, Waldbronn, Germany) managed by ChemStation software (B.04.03). Preparative HPLC for isolation was performed using a Gilson 321 pump (Gilson, Middleton, WI, USA) and Waters 2487 detector (Waters, Milford, MA, USA). For the structural identification of isolated compounds, a Waters Acquity UPLC system (Waters, Milford, MA, USA) equipped with an electrospray ionization source interfaced to an Advion expression CMS mass spectrometer (Advion, Ithaca, NY, USA) and an AVANCE III 400 spectrophotometer (Bruker, Ettlingen, Germany) were used. HPLC-grade acetonitrile and water were obtained from Daejung Chemical (Siheung, Republic of Korea), while analytical-grade formic acid was purchased from Sigma Aldrich (St. Louis, MO, USA). All additional organic solvents utilized for sample extraction and CPC separation were obtained from Daejung Chemical (Siheung, Republic of Korea).

### 3.2. Plant Material and Preparation of the Cp Extract

The plant materials of *Cp* were purchased from an oriental drug store (Kwang Myung Dang Co., Ulsan, Republic of Korea) and were authenticated by one of the corresponding authors (C.Y.K.). A voucher specimen (specimen no. HYUP-CP-001) has been deposited in the Pharmacognosy Laboratory of the College of Pharmacy, Hanyang University. The powdered *Cp* (482.0 g) was subjected to three extractions with 5 L of methanol using an ultrasonic apparatus at room temperature. The resulting extract was then concentrated in vacuo, yielding a methanol extract (33.3 g, 6.9%). This extract was suspended in water and successively fractionated with equal volumes of HX. The obtained HX fraction (12.0 g) was stored in a refrigerator for CPC separation.

### 3.3. Preparation of the Two-Phase Solvent System

The two-phase solvent system was determined based on the suitable partition coefficients (0.5 < *K* < 2.0) of the target compounds, tested across various solvent systems composed of pre-equilibrated *n*-hexane:ethanol:water. To achieve this, 10–15 mg of crude extract was dissolved in 1.8 mL of each phase of the immiscible two-phase solvent system in separate tubes. Each tube was vigorously shaken for several minutes and then allowed to reach equilibrium. Subsequently, equal volumes of the upper and lower phases were analyzed by HPLC at 254 nm. In this analysis, the upper organic phase served as the mobile phase, while the lower aqueous phase functioned as the stationary phase. The partition coefficient (*K* value) for each compound was determined by calculating the ratio of the peak area in the upper phase to that in the lower phase.

### 3.4. CPC and Preparative HPLC Separation of the HX Fraction

Based on the appropriate *K* values, CPC separation was carried out using a two-phase solvent system consisting of *n*-hexane–ethanol–water (10:7:3, *v*/*v*/*v*). The biphasic solvent system was prepared by thorough mixing and complete equilibration of the upper and lower phase solvents. The CPC rotor was initially filled with the lower layer as the stationary phase. Subsequently, the rotation speed of the rotor was increased to 1100 rpm, and the upper layer, serving as the mobile phase, was introduced into the rotor in ascending mode with a flow rate of 10 mL/min. Once the CPC rotor attained equilibrium in the immiscible biphasic state (with 730 mL of the stationary phase out of a total rotor volume of 1000 mL, at a pressure of 82 bar), 4.7 g of HX fraction dissolved in a 14 mL mixture of the upper and lower phases was introduced into the CPC system. The effluent was monitored at wavelengths of 254 and 280 nm. Every peak fraction was collected at 25 mL per tube and separated according to the elution profile to yield three CPC peak fractions I–III. The fractions were dried and analyzed by HPLC. As a result, compounds **1** and **2** were obtained with high purity; compound **1** (190.1 mg) from CPC peak fraction III; compound **2** (914.9 mg) from CPC peak fraction II. For the further purification of the CPC peak fraction I, preparative HPLC was conducted with a Cosmosil C18 column (5 µm, 20.0 × 150 mm) to purify compound **3** (15.0 mg).

### 3.5. HPLC Analysis

The extract and the CPC peak fraction were analyzed by HPLC equipped with a Hector C18 column (5 µm, 4.6 × 250 mm). The mobile phase was composed of 0.1% formic acid in both water (A) and acetonitrile (B), with the gradient elution conditions as follows: 0–5 min (25–50% B), 5–10 min (50–70% B), 10–22 min (70% B), 22–25 min (70–100% B), and 25–30 min (100% B). The injection volume was 10 µL, and the flow rate was kept at 1 mL/min. The diode array detector recorded the UV spectrum from 210–400 nm, and the chromatogram was monitored at 254 nm.

### 3.6. Structural Elucidation

The structural elucidation of the isolated compounds was carried out by ESI-MS and NMR analysis. NMR spectra were obtained in chloroform-*d* and processed using MestReNova (Mnova) 9.0 software (Mestrelab Research Inc., Santiago de Compostela, Spain). Additionally, the conditions for the ESI-MS spectra were as follows: positive ion mode; mass range of *m/z* 100–1200; capillary temperature set to 200 ℃; capillary voltage at 150 V; source voltage offset of 30; source voltage span of 10; source gas temperature at 150 ℃; and ESI voltage of 3500 V. The ^1^H-, ^13^C-NMR, and ESI-MS spectra of isolated compounds **1**–**3** are shown in Supplementary Materials.

### 3.7. Measurement of PrP^Sc^ Levels and Cytotoxicity in ScN2a Cells

Curcumenone (**1**), curcumenol (**2**), and furanodienone (**3**) (purity ≥ 98%) were purchased from Chemfaces (Wuhan, China). The compounds were dissolved in dimethyl sulfoxide and used for the assays. The cytotoxicity of the compounds was determined by MTT assay as previously described [32]. Briefly, ScN2a cells were cultured with the compounds for 4 days, and the level of formazan formation from MTT agents (Sigma, St. Louis, MO, USA) by cellular enzymes was measured with the Infinite M200Pro Multimode Reader (Tecan, Männedorf, Switzerland) at 570 nm. The level of PrP^Sc^ in ScN2a cells was analyzed as previously described [13]. Briefly, ScN2a cells were cultured with the compounds for 4 days at non-cytotoxic concentrations (>90% of cell viability), and proteinase K (PK) (Roche, Basel, Switzerland)-resistant PrP^Sc^ in cell lysates was analyzed by Western blotting using mouse monoclonal anti-PrP antibody 6D11 (Biolegend, San Diego, CA, USA). Separately, β-actin, as a loading control marker, was detected using mouse monoclonal anti-β-antibody AC-15 (Santa Cruz Biotechnology, Dallas, TX, USA). Densitometry of Western blots was performed using Gene Tools 4.1 software (Syngene, San Diego, CA, USA). Student’s *t*-test was used for statistical analysis.

## 4. Conclusions

This study presents the successful establishment of a bioactivity-guided separation method using CPC to isolate and purify active compounds from effective *Cp* extract against prion diseases. Following the activity screening of solvent fractions from the extract, the HX fraction was revealed to retain anti-prion activity exhibited by the crude extract. The application of CPC makes it possible to achieve the effective separation and high-yield purification of major compounds from the HX fraction in a single step. Structural elucidation by spectroscopic analysis identified major compounds as curcumenone (**1**), curcumenol (**2**), and furanodienone (**3**). Estimation of the anti-prion activity of these compounds confirmed curcumenol (**2**) and furanodienone (**3**) are the active ones, showing significant efficacy to reduce PrP^Sc^ accumulation in a cell culture model of prion disease. Further research in animal models is needed to explore whether these compounds are able to modify the course of disease and ameliorate neuropathological and molecular biomarkers associated with prion disease. Enhancing the potential for therapeutic applications, this finding contributes to ongoing efforts to develop treatments for prion diseases. Additionally, the current study emphasizes the importance of natural product-based research for drug discovery to combat prion disease.

## Figures and Tables

**Table 1:** The *K*-values of three main compounds of the HX fraction of *Cp* extract.

*n*-HX:ethanol:water (*v*/*v/v*)	*K*-Values		
	Curcumenone (1)	Curcumenol (2)	Furanodienone (3)
10:9:1	0.27	0.44	0.81
10:8:2	0.20	0.41	1.18
10:7:3	0.26	0.58	2.14

## Data Availability

All data generated or analyzed during this study are included in this published article and its Supplementary Materials.

## Supplementary Materials

The following supporting information can be downloaded at https://www.mdpi.com/article/10.3390/molecules29174034/s1, Table S1: Fraction weights and percent ratio of solvent-fractionated *Cp* extract; Table S2: The partition coefficients (*K*) of target compounds of the HX fraction in different solvent systems; Figure S1: HPLC chromatogram at 254 nm of the HX fraction from *Cp* extract; Figure S2: ^1^H-NMR (400 MHz) spectrum of curcumenone (**1**) in chloroform-*d*; Figure S3: ^13^C-NMR (100 MHz) spectrum of curcumenone (**1**) in chloroform-*d*; Figure S4: ^1^H-NMR (400 MHz) spectrum of curcumenol (**2**) in chloroform-*d*; Figure S5: ^13^C-NMR (100 MHz) spectrum of curcumenol (**2**) in chloroform-*d*; Figure S6: ^1^H-NMR (400 MHz) spectrum of furanodienone (**3**) in chloroform-*d*; Figure S7: ^13^C-NMR (100 MHz) spectrum of furanodienone (**3**) in chloroform-*d*; Figure S8: ESI-MS spectrum of isolated compounds; Figure S9: Cytotoxicity of identified compounds from the HX fraction.
