# Supplementary material for: Isolation of Anti-Prion Compounds from Curcuma phaeocaulis Valeton Extract
Source: Molecules. 2024 Aug 26;29(17):4034. doi: 10.3390/molecules29174034 (PMC11397528; doi:10.3390/molecules29174034)
Supplement: Supplementary file 1 [file molecules-29-04034-s001.zip › molecules-3097068-supplementary.pdf]

Supplementary Materials

Isolation of Anti-Prion Compounds from *Curcuma phaeocaulis* Valeton Extract

Jaehyeon Kim†, Hakmin Lee†, Hye Mi Kim†, Ji Hoon Kim, Sanghoon Byun,

Sungeun Lee, Chul Young Kim* and Chongsuk Ryou*

Department of Pharmacy, College of Pharmacy and Institute of Pharmaceutical Science and Technology, Hanyang University ERICA, Ansan, Gyeonggi-do 15588, Republic of Korea; rlawoguses@hanyang.ac.kr (J.K.); gkrals92@hanyang.ac.kr (H.L.); hyemi586@hanyang.ac.kr (H.M.K.); gg890718@gmail.com (J.H.K.); chok9916@hanyang.ac.kr (S.B.); guranye@hanyang.ac.kr (S.L.); chulykim@hanyang.ac.kr (C.Y.K.); cryou2@hanyang.ac.kr (C.R.)

* Corresponding authors: Chul Young Kim; Chongsuk Ryou

E-mail address: chulykim@hanyang.ac.kr (C.Y.K.); cryou2@hanyang.ac.kr (C.R.)

† These authors contributed equally to this work.

Table S1. Fraction weights and percent ratio of solvent fractionated *Cp* extract.

Fractions	Weight (g)	Weight ratio (%)	Calculated conc. (μg/ml)
CpV Ext	4.26	100	50
HX	1.85	43.4	21.7
EA	1.72	40.4	20.2
ED	0.13	3.1	1.5
DW	0.56	13.1	6.6

Table S2. The partition coefficients (*K*) of target compounds of HX fraction in different solvent systems.

**Solvent system**	**Volume ratio**	***K*-values**				
		Peak **1**	Peak **2**	Peak **3**	Peak **4**	Peak **5**
H:M:W	10:9:1	0.17	0.23	0.84	1.00	1.08
	10:8:2	0.25	0.36	1.82	2.17	2.37
	10:7:3	0.47	0.67	4.53	5.18	5.75
H:I:W	10:8:2	0.68	1.24	1.52	1.53	1.56
	10:7:3	0.75	1.86	2.62	2.64	2.71
	10:6:4	1.11	2.18	2.63	2.67	2.64
H:E:W	10:9:1	0.27	0.44	0.74	0.80	0.81
	10:8:2	0.20	0.41	1.03	1.16	1.18
	10:7:3	0.26	0.58	1.87	2.06	2.14
H:A:W	10:9:1	0.10	0.17	0.46	0.47	0.57
	10:8:2	0.15	0.24	0.71	0.73	0.87
	10:7:3	0.24	0.37	1.43	1.45	1.72
H:I:M:W	9:1:9:1	0.16	0.23	0.72	0.83	0.92
	9:1:8:2	0.20	0.32	1.38	1.58	1.79
	8:2:8:2	0.19	0.32	1.11	1.29	1.41
H:EA:E:W	9:1:9:1	0.33	0.50	0.77	0.83	0.84
H:EA:M:W	9:1:9:1	0.19	0.26	0.78	0.89	0.98

The *K*-value is defined as the peak area of the compounds in upper stationary phase divided by the peak area of those in the lower mobile phase. Each peak corresponds to those observed in the HPLC chromatogram of the HX fraction, as depicted in Figure S1. H: *n*-hexane, M: methanol, W: water, I: isopropanol, E: ethanol, A: acetonitrile, EA: ethyl acetate

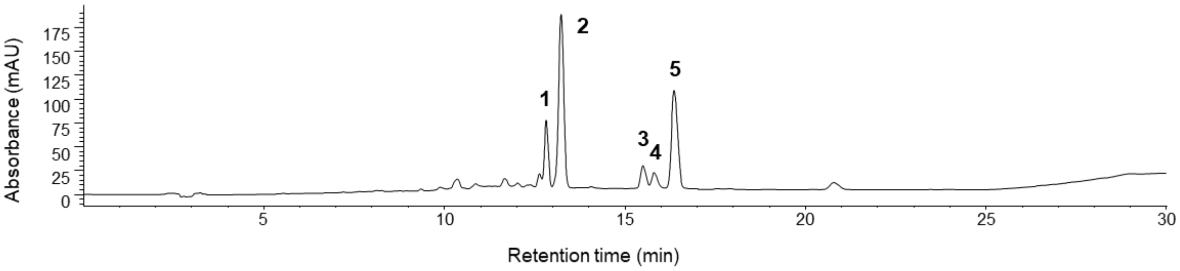

Figure S1. HPLC chromatogram at 254 nm of HX fraction from *Cp* extract.

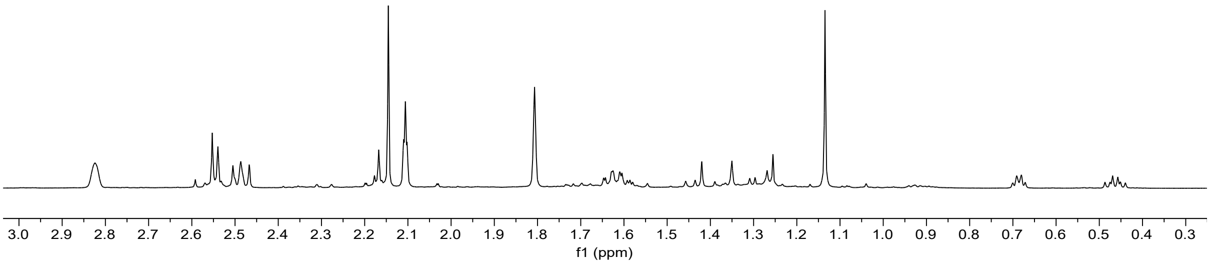

Figure S2. ^{1}H-NMR (400 MHz) spectrum of curcumenone (**1**) in chloroform-*d*.

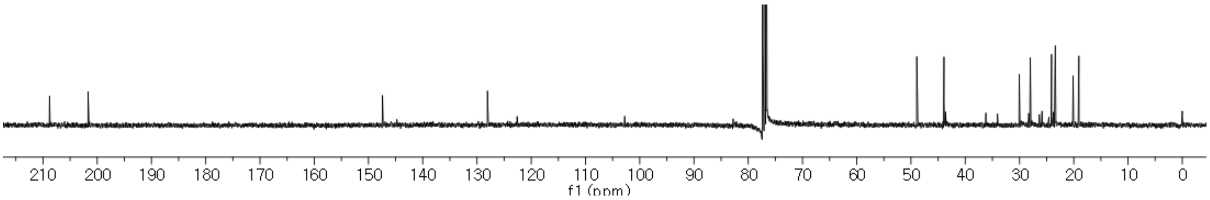

Figure S3. ^{13}C-NMR (100 MHz) spectrum of curcumenone (**1**) in chloroform-*d*.

Curcumenone (1)

^{1}H NMR (400 MHz, Chloroform-*d*) δ 2.82 (s, 2H, H-7), 2.55 (d, *J* = 5.2 Hz, 2H, H-10), 2.50 (m, 2H, H-4), 2.15 (s, 3H, H-15), 2.11 (t, *J* = 1.6 Hz, 3H, H-12), 1.81 (s, 3H, H-13), 1.62 (qd, *J* = 7.2, 2.0 Hz, 2H, H-3), 1.13 (s, 3H, H-14), 0.69 (q, *J* = 4.4 Hz, 1H, H-6), 0.46 (td, *J* = 7.2, 4.7 Hz, 1H, H-2); ^{13}C NMR (100 MHz, Chloroform-*d*) δ 208.8 (C-5), 201.7 (C-9), 147.4 (C-11), 128.1 (C-8), 48.9 (C-10), 43.9 (C-4), 30.1 (C-2), 28.0 (C-7), 24.2 (C-6), 24.1 (C-2), 23.5 (C-3), 23.4 (C-12), 23.4 (C-13), 20.1 (C-1), 19.1 (C-14).

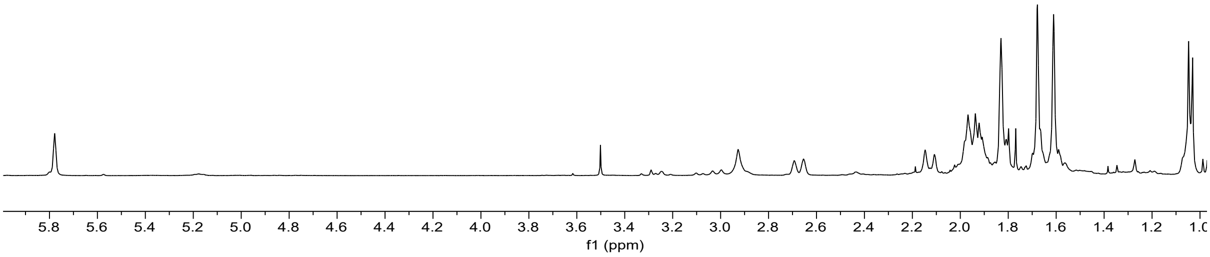

Figure S4. ^{1}H-NMR (400 MHz) spectrum of curcumenol (**2**) in chloroform-*d*.

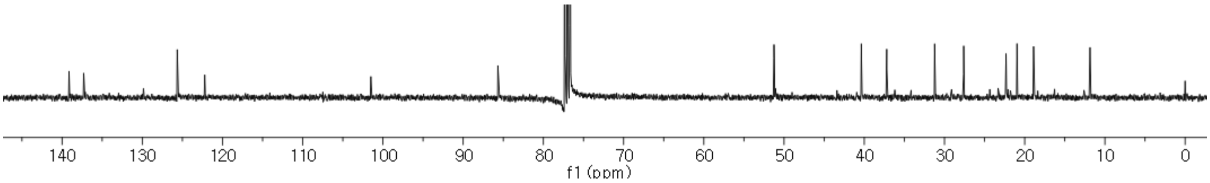

Figure S5. ^{13}C-NMR (100 MHz) spectrum of curcumenol (**2**) in chloroform-*d*.

Curcumenol (2)

^{1}H NMR (400 MHz, Chloroform-*d*) δ 5.78 (s, 1H, H-9), 2.93 (s, 1H, H-17), 2.67 (d, *J* = 15.6 Hz, 1H, H-6), 2.13 (d, *J* = 15.6 Hz, 1H, H-6), 1.93 (m, 6H, H-1, 2, 3, 4), 1.83 (s, 3H, H-12), 1.68 (s, 3H, H-13), 1.61 (s, 3H, H-15), 1.04 (d, *J* = 6.8 Hz, 3H, H-14); ^{13}C NMR (100 MHz, Chloroform-*d*) δ 139.1 (C-7), 137.3 (C-10), 125.6 (C-9), 122.2 (C-11), 101.5 (C-8), 85.7 (C-5), 51.3 (C-1), 40.4 (C-4), 37.2 (C-6), 31.2 (C-3), 27.6 (C-2), 22.3 (C-12), 21.0 (C-15), 18.9 (C-13), 11.9 (C-14).

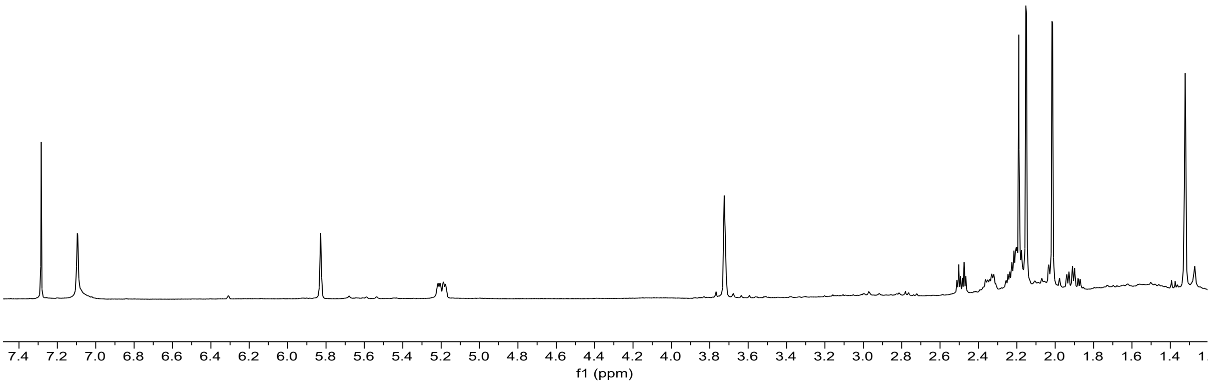

Figure S6. ^{1}H-NMR (400 MHz) spectrum of furanodienone (**3**) in chloroform-*d*.

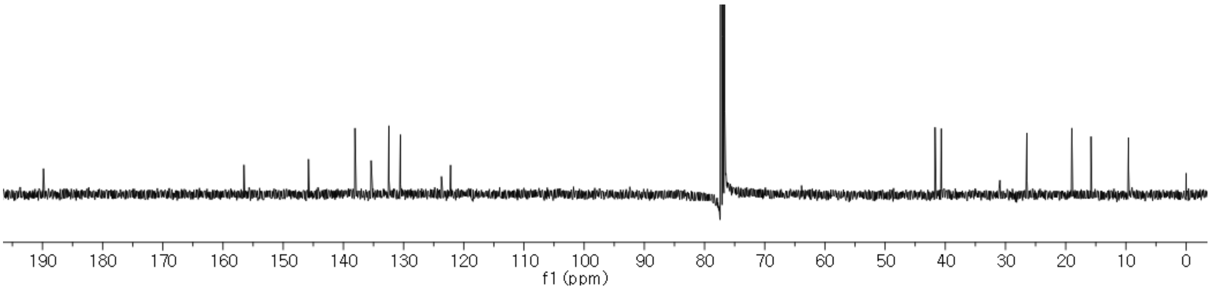

Figure S7. ^{13}C-NMR (100 MHz) spectrum of furanodienone (**3**) in chloroform-*d*.

Furanodienone (3)

^{1}H NMR (400 MHz, Chloroform-*d*) δ 7.10 (s, 1H, H-12), 5.20 (dd, *J* = 11.6, 4.4 Hz, 1H, H-5), 3.72 (s, 2H, H-9), 2.67 (dt, *J* = 11.6, 4.0 Hz, 1H, H-3), 2.34 (m, 1H, H-2), 2.33 (m, 1H, H-2), 2.15 (s, 3H, H-15), 2.02 (s, 3H, H-14), 1.90 (td, *J* = 11.6, 4.4 Hz, 1H, H-3), 1.32 (s, 3H, H-15). ^{13}C NMR (100 MHz, Chloroform-*d*) δ 189.8 (C-6), 156.5 (C-8), 145.8 (C-4), 138.1 (C-12), 135.4 (C-10), 132.4 (C-5), 130.5 (C-1), 123.7 (C-11), 122.2 (C-7), 41.7 (C-3), 40.7 (C-9), 26.4 (C-2), 19.0 (C-14), 15.8 (C-15), 9.6 (C-13).

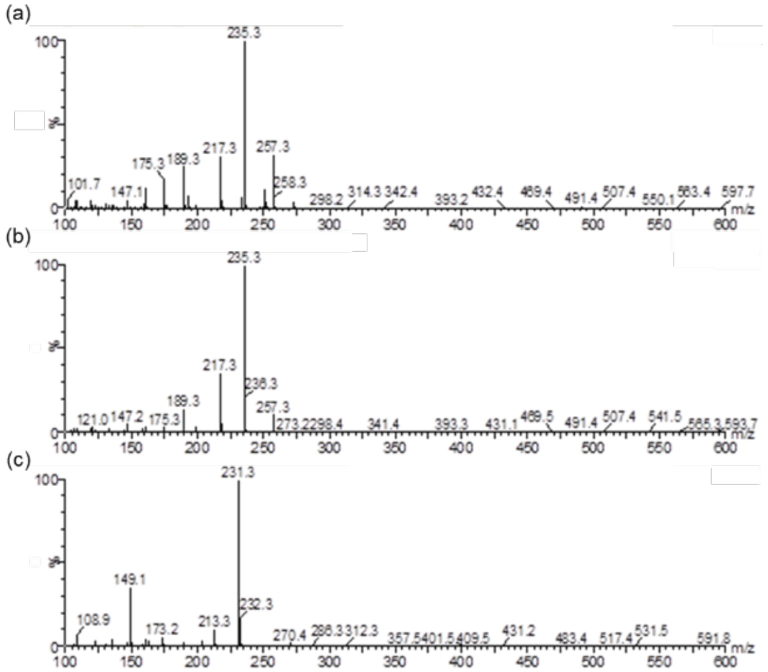

Figure S8. ESI-MS spectrum of isolated compounds. (a) curcumenone (**1**, *m*/*z* 235.3 [M+H]+), (b) curcumenol (**2**, *m*/*z* 235.3 [M+H]+), and (c) furanodienone (**3**, *m*/*z* 231.3 [M+H]+)

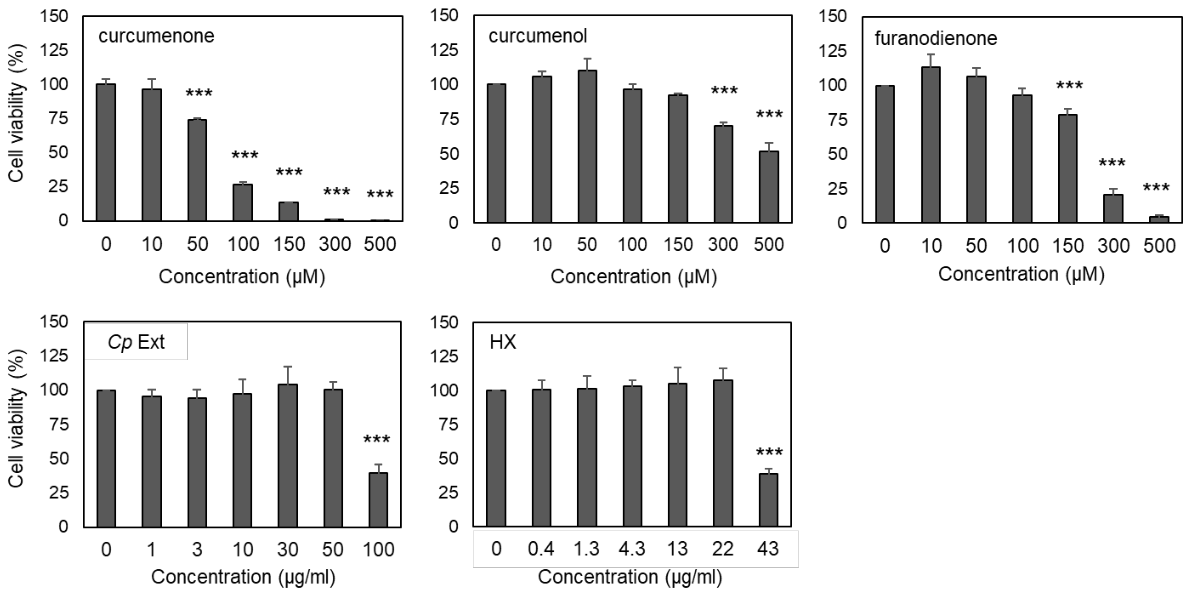

Figure S9. Cytotoxicity of identified compounds from HX fraction. MTT assay was conducted using ScN2a cells incubated with various concentrations of compounds, crude extract, and HX fraction. ***, *p*-value < 0.001 (n=3).
